# Combining Clickstream Analyses and Graph-Modeled Data Clustering for Identifying Common Response Processes

**DOI:** 10.1007/s11336-020-09743-0

**Published:** 2021-02-05

**Authors:** Esther Ulitzsch, Qiwei He, Vincent Ulitzsch, Hendrik Molter, André Nichterlein, Rolf Niedermeier, Steffi Pohl

**Affiliations:** 1 Educational Measurement, IPN – Leibniz Institute for Science and Mathematics Education, Olshausenstraße 62, 24118 Kiel, Germany; 2 Educational Testing Service, Princeton, USA; 3 Technische Universität Berlin, Berlin, Germany; 4 Freie Universität Berlin, Berlin, Germany

**Keywords:** action sequences, response times, complex problem solving, cluster editing

## Abstract

Complex interactive test items are becoming more widely used in assessments. Being computer-administered, assessments using interactive items allow logging time-stamped action sequences. These sequences pose a rich source of information that may facilitate investigating how examinees approach an item and arrive at their given response. There is a rich body of research leveraging action sequence data for investigating examinees’ behavior. However, the associated timing data have been considered mainly on the item-level, if at all. Considering timing data on the action-level in addition to action sequences, however, has vast potential to support a more fine-grained assessment of examinees’ behavior. We provide an approach that jointly considers action sequences and action-level times for identifying common response processes. In doing so, we integrate tools from clickstream analyses and graph-modeled data clustering with psychometrics. In our approach, we (a) provide similarity measures that are based on both actions and the associated action-level timing data and (b) subsequently employ cluster edge deletion for identifying homogeneous, interpretable, well-separated groups of action patterns, each describing a common response process. Guidelines on how to apply the approach are provided. The approach and its utility are illustrated on a complex problem-solving item from PIAAC 2012.

## Introduction

Interactive items in low-stakes large-scale assessments are designed to provide authentic tasks and, as such, to better reflect what examinees know and are able to do than traditional test items can (Goldhammer, Naumann, & Keßel, 2013). Such kind of items is used, for example, in the Problem Solving in Technology-Rich Environments (PSTRE) domain in the Programme for the International Assessment of Adult Competencies (PIAAC, OECD, 2013) and the collaborative problem solving domain in the Programme for International Student Assessment (PISA, OECD, 2017). Understanding response processes to interactive tasks is paramount for assessing whether these indeed capture the construct to be measured. As noted in the Standards for Educational and Psychological Testing “construct interpretations oftentimes involve more or less explicit assumptions about the cognitive processes engaged” (American Educational Research Association, American Psychological Association, & National Council on Measurement in Education and Joint Committee on Standards for Educational and Psychological Testing, 2014, p. 15). Therefore, “theoretical and empirical analyses of the response processes” (American Educational Research Association et al., 2014, p. 15) are recommended for assessing whether the response processes applied by examinees fit with the interpretation of the construct to be measured.

**Fig. 1:**
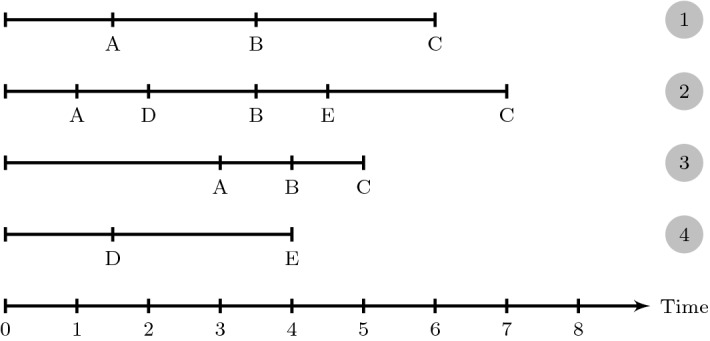
Schematic representation of time-stamped action sequences for four hypothetical examinees

Being computer-administered, assessments using interactive items allow logging time-stamped action sequences. These sequences, illustrated schematically in Fig. 1, document both the particular actions executed and the time required for their execution. Various approaches exist that leverage action sequence data for investigating how examinees interact with interactive items (e.g., He & von Davier, 2015; Qiao & Jiao, 2018; Tang, Wang, He, Liu, & Ying, 2020; Tang, Wang, Liu, & Ying, 2020). The associated timing data, however, have mainly been considered on an aggregate level (e.g., time spent on task as opposed to time required for the single actions executed for completing the task), if at all. Since differences in timing can be indicative of different underlying cognitive processes even if the same actions are performed, considering action sequences jointly with the time elapsed between the actions constituting the sequences has vast potential to support a more fine-grained assessment of examinees’ interactions with interactive items. For instance, it may support detecting parts of response processes that are more time consuming for examinees, e.g., due to being cognitively more challenging.

To motivate the use of time-stamped action sequences for a more in-depth assessment of response processes, we consider action patterns for Examinees 1 and 3 in Fig. 1, performing the same action sequence within a comparable amount of time. However, while Examinee 1 executed his or her first action rather quickly, Examinee 3 initially required more time but then performed all actions in quick succession. These differences in time elapsing between actions may mark different response processes. Examinee 3 might have spent long time for carefully planning how to approach the task, while Examinee 1 might have planned on-the-go, resulting in a shorter time to first action but longer time between subsequent actions required for planning the next step. Such differences cannot be uncovered by solely considering action sequences or time spent on the whole task but needs considering action-level timing data.

In this article, we aim at making use of the whole of information contained in time-stamped action sequences and provide an approach that jointly considers action sequences and the corresponding sequence of time elapsed between the actions for identifying common response processes. For doing so, we combine data mining techniques originally developed for the analysis of clickstream data with graph-modeled data clustering.

The remainder of this article is structured as follows: First, we provide an overview of previous approaches for making use of action sequences, timing data, or both. We then present an approach for identifying common response processes that is based on the information contained in time-stamped action sequences. We illustrate the insights that can be gained on the basis of this approach by applying it to a PIAAC PSTRE task.

### Using Action Sequences for Investigating Response Processes

Making use of action sequences for investigating examinees’ interactions with interactive tasks is a rapidly growing stream of research. One of the main challenges for making use of action sequences is how to meaningful aggregate this usually enormous amount of data (von Davier, Khorramdel, He, Shin, & Chen, 2019).

In the case that subject-matter theory exists on how examinees approach interactive tasks, theory-derived indicators can be constructed (e.g., whether examinees employed a certain solution strategy or not). These can then be related to other variables of interest (Greiff, Niepel, Scherer, & Martin, 2016; Greiff, Wüstenberg, & Avvisati 2015) or even be employed as indicators in measurement models (LaMar, 2018). However, given that action sequence data are usually complex, reflecting the wide diversity of human behavior (Tang, Wang, Liu, & Ying, 2020), most of the approaches for such data are exploratory in nature.

Visual approaches aim at providing graphical frameworks for depicting action sequence data that assist discovering meaningful patterns in the data, e.g., important actions or pathways (Vista, Care, & Awwal, 2017; Zhu, Shu, & von Davier, 2016). Similar objectives have been pursued by employing data mining techniques for identifying single actions or subsequences (*n*-grams) that are associated with success or failure on an interactive task or that differentiate between different proficiency groups (He & von Davier, 2015; 2016; Liao, He, & Jiao, 2019; Qiao & Jiao, 2018; Stadler, Fischer, & Greiff, 2019).

Another common approach for detecting patterns in action sequence data for the purpose of investigating examinees’ interactions with interactive tasks is to compress the information contained in differences between any two action sequences into distance measures. In this context, distance measures can either be defined to describe how action sequences differ from each other (Tang, Wang, He, et al., 2020) or with regard to expert-defined optimal strategies (Hao, Shu, & von Davier, 2015; He, Borgonovi, & Paccagnella, 2019a) and are usually derived by drawing on techniques from natural language processing, such as the Levenshtein edit distance (Hao et al., 2015) or longest common subsequences (LCSs; He, Borgonovi, & Paccagnella, 2019a; Sukkarieh, von Davier, & Yamamoto, 2012), or from the field of clickstream analysis (Tang, Wang, He, et al., 2020). The information contained in such distance measures can then be further used in employing exploratory dimensionality reduction techniques such as principal component analysis and hierarchical clustering (Eichmann, Greiff, Naumann, Brandhuber, & Goldhammer, 2020; Hao et al., 2015), or multidimensional scaling (Tang, Wang, He, et al., 2020). When distance from expert-defined optimal strategies is assessed, distance measures can be related to other variables of interest, for example, proficiency. This allows assessing whether similarity with optimal strategies indeed contains information on examinees’ proficiency levels (He, Borgonovi, & Paccagnella, 2019a).

Recently, new approaches have been developed that draw on machine learning techniques for assessing response processes by complexity reduction. Recurrent neural networks, for instance, have successfully been applied for extracting latent features for parsimoniously describing response processes (Tang, Wang, Liu, & Ying, 2020) or for breaking down individual processes into a sequence of subprocesses (Wang, Tang, Liu, & Ying, 2020).

### Using Timing Data for Investigating Response Processes

Using timing data for inferring the nature of cognitive processes has a long tradition in psychology and is a key element for drawing inferences about cognitive and behavioral processes in a variety of paradigms and theoretical frameworks (see De Boeck & Jeon, 2019; Kyllonen & Zu, 2016, for overviews). These are built on the rationale that differences in timing data are indicative of qualitative or quantitative differences in cognitive processes that differ in the time required for their execution. A prominent example for such differences is the distinction between solution and rapid guessing behavior in the context of multiple-choice items, where both processes can result in choosing the same answer on a multiple-choice item but are likely to be associated with rather different response times (Wise, 2017). In the context of traditional test items (i.e., items with a multiple-choice or open-response format) there is a rich body of research using timing data for better understanding response behavior, e.g., by assessing how examinees allocate their time during the assessment (e.g., Fox & Marianti, 2016) or for detecting differences in response processes (e.g., Molenaar, Oberski, Vermunt, & De Boeck, 2016; Partchev & De Boeck, 2012; Ulitzsch, von Davier, & Pohl, 2019; 2020 ; Wang & Xu, 2015; Wang, Xu, Shang, & Kuncel, 2018; Weeks, von Davier, & Yamamoto, 2016).

In the context of interactive tasks, research focusing on timing data has mainly focused on item-level time, for instance, to investigate how time spent on an item is related to proficiency (Goldhammer et al., 2014; Naumann & Goldhammer, 2017; Scherer, Greiff, & Hautamäki, 2015). There are, however, some exceptions. Stelter, Goldhammer, Naumann, and Rölke (2015) assessed time spent on pre-selected, automatable subtasks such as drag-and-drop events or setting a bookmark via the toolbar of a browser. The authors argued that shorter time spent on automatable subtasks indicates a higher degree of automation of the procedural knowledge needed to execute these subtasks. In support of this, the authors showed that examinees with shorter time spent on automatable subtasks were more likely to succeed on PIAAC PSTRE tasks, indicating higher levels of proficiency. In a similar vein, Albert and Steinberg (2011) assessed whether planning time, defined as the time elapsed from beginning the task until performing the first action, is related to successful task completion. Using data from the PISA 2012 problem solving domain, Eichmann, Goldhammer, Greiff, Pucite, and Naumann (2019) built on that work and derived indicators that allow depicting planning behavior in greater detail. The authors considered (a) the longest time interval elapsed between actions, conceptualized as the longest planning interval, (b) the time elapsed until the longest planning interval occurred as a measure for the time when (most of) the planning takes place, and c) the variance of times elapsed between any two successive actions, giving the variation in planning time. Both Albert and Steinberg (2011) and Eichmann et al. (2019) could show that planning time is beneficial for successful task completion. It is noted that the objective of these studies was to assess the predictive power of features derived from action-level times for successful task completion. They do, however, not allow for disentangling and describing different response processes in terms of the types and order of performed actions.

### Combining Information from Action Sequences and Timing Data

Few approaches exist that consider both information from action sequences and timing data for the purpose of investigating examinees’ interactions with interactive tasks. The majority of these approaches considers timing data only on the item-level, that is, takes into account the total time spent on an item rather than the time taken for each performed action (time to action).

In a confirmatory approach, De Boeck and Scalise (2019) considered both action sequences and timing data as aggregated variables, employing the number of actions, total time spent on the item, and performance as indicators of a three-dimensional latent variable model. This framework allows assessing how the number of actions taken and the time required for solving a task relate to proficiency.

Exploratory approaches jointly considering information on actions and timing are predominantly aimed at identifying groups differing in their interaction with the tasks. To that end, He, Liao, and Jiao (2019b) used *k*-means clustering based on actions, the length of action sequences, and time spent on the item. Xu, Fang, Chen, Liu, and Ying (2018) employed latent class analyses based on the frequency of recurrent actions and time spent on the item. Their approach allowed the detection of classes differing in the degree of efficiency of solution behavior—defined in terms of how often the same actions were performed—and to assess differences in time spent on the item between the classes.

Another approach to analyze timing data in addition to action sequences is to treat time spent on task as a covariate that can be considered for further investigation of identified response processes. Wang et al. (2020), for instance, assessed whether the time spent on a task is related to employing different solution strategies.

One exception considering action sequences jointly with non-aggregated timing data is the work conducted by Chen, Li, Liu, and Ying (2019), who presented an exploratory event history approach to simultaneously predict total time spent on the item as well as the final response. Various features derived from action sequences (such as action frequencies or indicators of whether a certain action has previously been executed) were used as predictors. By considering features derived from action sequences in continuous time, the approach takes the timing of actions into account. Note that the objective of this approach is closely related to approaches that aim at identifying key actions and subsequences that are relevant for success on an interactive item (see He & von Davier, 2015; Liao et al., 2019; Qiao & Jiao, 2018). It does, as such, not aim for describing differences in response processes, considering the type and timely order of action sequences.

In sum, there is a rich and rapidly growing body of research aiming to make use of the information contained in time-stamped action sequences, either for assessing the predictive power of actions and timing for successful task completion or for investigating differences in response processes. The latter class of approaches, however, so far has considered timing data only on the aggregate item-level. By considering aggregated features such as time spent on task rather than time elapsed between actions, these previous approaches neglect that examinees may differ in the time—and, as such, the underlying cognitive processes—required for executing specific subprocesses. Therefore, the aim of this article is to develop a new approach that can make use of the whole of information contained in time-stamped action sequences for a more in-depth investigation of the behavioral processes underlying task completion.

## Proposed Method

We propose a two-step approach that integrates tools from clickstream analyses and graph-modeled data clustering with psychometrics and combines action sequences and action-level times into one analysis framework. We leverage the information contained in action patterns as given by action sequences and action-level times (a) to determine the degree of similarity between action patterns and (b) to identify common response processes. For identifying subgroups of persons with similar action patterns, we propose performing cluster editing—a graph-modeled data clustering technique—on the similarity measures.

In the following, we first present two similarity measures considering action sequences and times to action that vary in their degree of sensitivity to time-wise differences. We then introduce cluster editing as a mean for identifying common response processes given by homogeneous subgroups with similar action patterns. An existing integer linear programming (ILP) formulation of the cluster editing problem is explained.

### Required Data Structure

The employed similarity measure is based on action patterns in terms of action sequences and the associated times to action. Action patterns are represented as a *u*-length sequence of ordered pairs $\mathbf {p}_\mathrm{i} = [(a_{i1}, t_{i1}) (a_{i2}, t_{i2}) \ldots (a_{iu}, t_{iu})]$. Examinee *i*’s action sequence is given by ${\mathbf {a}}_{i} = \langle a_{i1}, a_{i2}, \ldots , a_{iu} \rangle$, with $a_{im}$ denoting the *m*th action executed by examinee *i*. The corresponding sequence of times to action is given by ${\mathbf {t}}_{i} = \langle t_{i1}, t_{i2}, \ldots , t_{iu} \rangle$, with $t_{im}$ giving the time to action associated with $a_{im}$, that is, the time that elapsed between performing action $a_{im-1}$ and action $a_{im}$. Hence, $t_{i1}$ is the time elapsed until the first action after examinee *i* is administered the item or exposed to the item interface.

### Determining Similarity of Action Patterns

For determining the degree of similarity between action patterns, we use and adapt a similarity measure presented by Banerjee and Ghosh (2001) that was originally developed in the context of analysis of clickstream data, aiming at investigating users’ interactions with websites. The rationale for drawing on this similarity measure is twofold. First, originating in clickstream analysis, the measure developed by Banerjee and Ghosh (2001) has been tailored to data types stemming from the real-life behavior typical interactive tasks aim to elicit. Second, and more importantly, the measure supports considering both the types and order of actions as well as the time elapsed in between. As will be shown, its sensitivity to differences in timing can easily be adjusted.

The similarity measure incorporates (a) the action sequences’ overlap in terms of their LCS, (b) the similarity of times to action in the area of the action sequences’ overlap, and (c) the importance of the area of overlap within the action sequences. The overlap of action sequences ${\mathbf {a}}_{i}$ and ${\mathbf {a}}_{j}$ of any two examinees *i* and *j* is determined by identifying their LCS, denoted by $\hbox {LCS}_{ij}$. The LCS represents the subsequence containing the maximum number of sequentially (but not necessarily adjacently) occurring actions that are shared by ${\mathbf {a}}_{i}$ and ${\mathbf {a}}_{j}$ (see He, Borgonovi, & Paccagnella, 2019a; Sukkarieh et al., 2012, for a detailed description). For identifying times to action associated with the actions constituting the LCS, for each pair of action sequences ${\mathbf {a}}_{i}$ and ${\mathbf {a}}_{j}$, two one-to-one functions $l^{i}()$ and $l^{j}()$ are obtained that map a particular index *m* of $\hbox {LCS}_{ij}$ to the corresponding indices $l^{i}(m)$ and $l^{j}(m)$ in the sequences ${\mathbf {a}}_{i}$ and ${\mathbf {a}}_{j}$, respectively. The times to action associated with the actions in $\hbox {LCS}_{ij}$ are given by ${\mathbf {t}}^{i}_{l^{i}(m)} = \langle t^{i}_{l^{i}(1)}, t^{i}_{l^{i}(2)}, \ldots , t^{i}_{l^{i}(|\hbox {LCS}_{ij}|)} \rangle$ and ${\mathbf {t}}^{j}_{l^{j}(m)} = \langle t^{j}_{l^{j}(1)}, t^{j}_{l^{i}(2)}, \ldots , t^{j}_{l^{j}(|\hbox {LCS}_{ij}|)} \rangle$, with $|\hbox {LCS}_{ij}|$ denoting the length of $\hbox {LCS}_{ij}$.

The similarity measure proposed by Banerjee and Ghosh (2001) weighs the similarity of times to action in the area of the LCS with the importance of the LCS within the action sequences. The similarity of times to action in the area of the LCS is defined as the average min–max similarity of all times to action associated with the actions constituting the LCS, formally

1 $$\begin{aligned} \text {sim}_{\hbox {LCS}_{ij}} = \frac{1}{|\hbox {LCS}_{ij}|} \sum _{m=1}^{|\hbox {LCS}_{ij}|} \frac{\min (t^{i}_{l^{i}(m)},t^{j}_{l^{j}(m)})}{\max (t^{i}_{l^{i}(m)},t^{j}_{l^{j}(m)})}. \end{aligned}$$

The importance of the actions constituting the LCS within the whole action sequence is given by the proportion of the total time spent on the LCS $T_{|\hbox {LCS}_{ij}|}^i$ on the total time spent on the task $T_\mathrm{tot}^i$. For calculating the average importance of the LCS across both sequences to be compared, Banerjee and Ghosh (2001) suggested to calculate the geometric mean of the actions’ importance within the sequences, that is

2 $$\begin{aligned} \text {imp}_{\hbox {LCS}_{ij}} = \left( \frac{T_{|\hbox {LCS}_{ij}|}^i}{T_\mathrm{tot}^i} \frac{T_{|\hbox {LCS}_{ij}|}^j}{T_\mathrm{tot}^j} \right) ^{\frac{1}{2}}. \end{aligned}$$

Finally, both average similarity of times to action and average importance are combined into a single similarity measure as

3 $$\begin{aligned} s_{ij} = \text {sim}_{\hbox {LCS}_{ij}} \text {imp}_{\hbox {LCS}_{ij}}. \end{aligned}$$

$s_{ij}$ takes values between 0 and 1, with 0 indicating no overlap between the action patterns, i.e., no LCS could be found, and 1 indicating exact similarity, of both ${\mathbf {a}}_{i}$ and ${\mathbf {a}}_{j}$ and ${\mathbf {t}}_{i}$ and ${\mathbf {t}}_{j}$ as constituting elements of ${\mathbf {p}}_i$ and ${\mathbf {p}}_{j}$.

#### Modified Similarity Measure

The similarity measure’s components can easily be adjusted. For instance, in its original form, the similarity measure proposed by Banerjee and Ghosh (2001) is sensitive to time-wise differences in single actions within the area of overlap of action sequences. Such sensitivity might not always correspond to research objectives and thus may yield less interpretable results. As an alternative that is less sensitive to time-wise differences in single actions, we propose to use the min–max similarity of time spent in the whole of area of overlap as a measure for the average similarity of times to action on $\hbox {LCS}_{ij}$. This modified measure aggregates timing data not on the item- but on the LCS-level. That is,

4 $$\begin{aligned} \text {sim}_{\hbox {LCS}_{ij}} = \frac{\min (\sum _{m=1}^{|\hbox {LCS}_{ij}|} t^{i}_{l^{i}(m)}, \sum _{m=1}^{|\hbox {LCS}_{ij}|}t^{j}_{l^{j}(m)})}{\max (\sum _{m=1}^{|\hbox {LCS}_{ij}|}t^{i}_{l^{i}(m)},\sum _{m=1}^{|\hbox {LCS}_{ij}|}t^{j}_{l^{j}(m)})}. \end{aligned}$$

As in the original similarity measure, the similarity measure $s_{ij}$ is then computed by weighing the average similarity of times to action on $\hbox {LCS}_{ij}$ with the average importance of $\hbox {LCS}_{ij}$ (see Eq. 3).

The modified $\text {sim}_{\hbox {LCS}_{ij}}$ takes the value 1 if examinees *i* and *j* spent the exact same amount of time on $\hbox {LCS}_{ij}$, regardless of individual time-wise differences in performed actions. Hence, the modified measure is better suited when researchers want to weigh similarities in action sequences stronger than similarities in the associated action-level times. Conversely, researchers should choose the original similarity measure when differences in timing of single actions defining the response process are of importance. An example for possible differences of interest are differences in automatable (e.g., drag-and-drop events) and non-automatable components (e.g., formulating an email) of the response process.

### Using Cluster Edge Deletion to Identify Subgroups with Similar Action Patterns

For identifying dominant response processes, given by subgroups for which action patterns within a subgroup are homogeneous but differ from action patterns outside the subgroup, we draw on cluster editing techniques (also referred to as correlation clustering; Bansal, Blum, & Chawla, 2004). Various clustering methods—*k*-means (see He, Liao, & Jiao, 2019b), density-based spatial clustering of applications with noise (DBSCAN, see Salles, Dos Santos, & Keskpaik, 2020), spectral clustering (see Trivedi, Pardos, Sárközy, & Heffernan 2011), nearest neighbor clustering (see Wollack & Maynes, 2017), or hierarchical clustering (see Hao et al., 2015) to name a few—have been employed in psychometric research for investigating response processes. Although quite common in biological applications (Böcker & Baumbach, 2013; Hartung & Hoos, 2015; Wittkop, Baumbach, Lobo, & Rahmann, 2007), cluster editing, however, has as of yet not been brought to the psychometric community’s attention. Cluster editing comes with the following advantages over other common clustering techniques that are especially useful in the context of clustering response processes. First, due to the manifold possibilities to approach a given task, researchers may have little domain knowledge for pre-determining features of the clustering outcomes such as the number of groups or the minimum cluster size. In contrast, by using cluster editing and related methods, possibly premature restrictions are avoided. In fact, cluster editing is described as an “agnostic learning” problem, trying to find the clustering which best describes the observed structures (Bansal et al., 2004). Second, researchers may find it challenging to interpret the clusters retrieved. Cluster editing methods may remedy this issue by (a) entailing an intuitive understanding of how clusters are determined and (b) allowing researchers to exert maximum control over what defines a cluster, thereby assisting to retrieve well-interpretable groups. Third, unlike some machine learning methods for assessing response processes (e.g., Tang, Wang, Liu, & Ying, 2020; Wang et al., 2020), cluster editing is especially well applicable under small sample conditions and may therefore pose a good method of choice when piloting items and investigating whether items indeed elicit the intended response processes. Illustratively, we focus on cluster edge deletion as a special case, use it to showcase the utility of cluster editing methods, and thereby add cluster editing to the psychometric toolbox.

Cluster editing problem statements aim at editing a weighted similarity graph into homogeneous, mutually exclusive subgroups by adding or deleting edges under the parsimony principle (Böcker & Baumbach, 2013). An edge-weighted undirected graph $G = (V,E,s)$ is a tuple where *V* denotes the vertices, $$E \subseteq \left( {\begin{array}{c}V\\ 2\end{array}}\right) $$ the edges, and *s*: $$\left( {\begin{array}{c}V\\ 2\end{array}}\right) \rightarrow {\mathbb {Q}}$$ the edge weights. Weighted cluster editing is the following problem: Given a weighted similarity graph $G=(V,E,s)$, find a minimum-weight set of edges to add or delete to transform *G* into cluster graph, that is, into a union of disjoint (i.e., unconnected) cliques. A clique is a subgraph where all vertices are adjacent to each other. Graphs in which every connected component is a clique are referred to as cluster graphs (Shamir, Sharan, & Tsur, 2004). In the cluster edge deletion problem statement, which is the focus of the present article, edges are only allowed to be deleted. Accordingly, the weighted cluster edge deletion problem statement is given by: Given a weighted similarity graph $G=(V,E,s)$, find a minimum-weight set of edges to delete to transform *G* into a cluster graph.

On an intuitive level, cluster editing techniques correspond to clustering objects in a given input graph. To uncover the clusters, cluster editing techniques edit the graph with minimum modifications such that the resulting graph only consists of cliques, i.e., distinct clusters (Böcker & Baumbach, 2013).

**Fig. 2:**
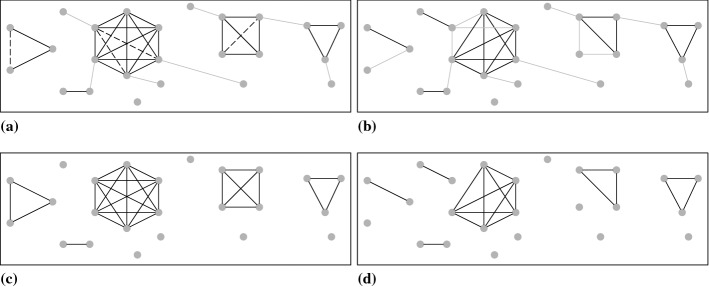
Cluster editing and cluster edge deletion instance before (**a**, **b**) and after editing (**c**, **d**); deleted edges in the input graph are marked in gray, dashed edges are inserted. The example is adapted from Böcker and Baumbach (2013)

Both cluster editing and cluster edge deletion on the same input graph are illustrated schematically in Fig. 2. For reasons of simplicity, we assume equal edge weights. Fig. 2a and b give the unedited input graph. Edges to be added or deleted when using cluster editing and cluster edge deletion, respectively, are marked in gray. Fig. 2c and d give the resulting output graphs for cluster editing and cluster edge deletion. As can be seen, cluster edge deletion yields cliques where all vertices are already connected to each other in the input graph. In contrast, by allowing for edge insertion and, as such, for connecting unconnected vertices, cluster editing may result in fewer and larger, but also less homogeneous cliques in the sense that not all vertices within a clique are required to be already connected to each other in the input graph.

For drawing on cluster editing techniques, we use the similarity measures described earlier to construct a weighted undirected similarity graph. Each vertex $v \in \{1,\dots ,N\}$ corresponds to one of the *N* action patterns, and the weight function $$s: \left( {\begin{array}{c}V\\ 2\end{array}}\right) \rightarrow {\mathbb {Q}}$$ assigns to each edge $e = \{i,j\}$ the respective similarity measure $s_{ij}$ as its edge weight. As usually (almost) all action patterns are pairwisely connected by a similarity measure $s_{ij} > 0$ (since actions like confirming an answer or proceeding to the next item are part of almost every action pattern), a threshold $\kappa$ is set as a lower bound of similarity necessary to indicate sufficient similarity of action patterns. If the similarity measure $s_{ij}$ is below the pre-defined threshold $\kappa$, the edge between *i* and *j* is not included in the constructed graph.

When performing cluster editing techniques on a graph containing information on similarities between action patterns, we consider each of the resulting cliques as one subgroup of action patterns. Isolated vertices with no connection to any other vertices in the graph pose cliques of size one. As such, these give unique, idiosyncratic action patterns. We focus on cluster edge deletion as a special case of cluster editing to enhance interpretability of results. When allowing for both edge insertion and deletion, dissimilar action patterns might be partitioned to the same clique if their action patterns are linked via similarities to other action patterns within the same clique. This might impede interpretation of the response process captured by such cliques. As we want to ensure that all action patterns constituting a clique have similarities of at least $\kappa$ with *all other* action patterns within that clique, we only allow for edges to be deleted, not to be added. Since when using cluster deletion two vertices only end up in the same clique if they were connected in the input graph, researchers can thus use the threshold $\kappa$ to incorporate their beliefs on the minimum degree of similarity between any two action patterns required to be considered as governed by a similar response process. Although this procedure does not imply that all action patterns within one clique overlap in exactly the same way, this guarantees a certain degree of homogeneity and thus interpretability of the captured response processes.

#### Integer Linear Programming Formulation of the Cluster Deletion Problem

Cluster deletion and cluster editing are NP-hard problems (see Bansal et al., 2004; Krivánek & Morávek, 1986; Shamir et al.,^2004^), meaning that most probably no polynomial time (i.e., fast) algorithm exists to solve these problems. However, by leveraging fixed-parameter algorithms or ILP, large instances could be solved in practice (Böcker & Baumbach, 2013). Given a weighted undirected Graph $G=(V,E,s)$, the following ILP formulation be can used for cluster edge deletion (adapted from Grötschel & Wakabayashi, 1989):

5 $$\begin{aligned} \text {Maximize} \nonumber \\&\displaystyle \sum _{ij \in E} s_{ij} \cdot x_{ij} \end{aligned}$$

6 $$\begin{aligned} \text {subject to} \nonumber \\&\displaystyle x_{ij} + x_{jk} - x_{ik} \le 1&\forall 1 \le i< j < k \le N \end{aligned}$$

7 $$\begin{aligned}&x_{ij} - x_{jk} + x_{ik} \le 1&\forall 1 \le i< j < k \le N \end{aligned}$$

8 $$\begin{aligned}&- x_{ij} + x_{jk} + x_{ik} \le 1&\forall 1 \le i< j < k \le N \end{aligned}$$

9 $$\begin{aligned}&x_{ij} \in \{0,1\}&\forall \{i,j\} \in E \end{aligned}$$

10 $$\begin{aligned}&x_{ij} = 0&\forall \{i,j\} \notin E \end{aligned}$$

Here the variable $x_{ij}$ corresponds to the edge $\{i,j\}$ between vertices *i* and *j*. The variable $s_{ij}$ gives the edge weight, i.e., the similarity of action patterns of examinees *i* and *j*. As usual, *E* denotes the set of edges of the input graph. We have $x_{ij}=1$ if $\{i,j\}$ is an element of the solution (meaning that it is in the input graph and is not deleted in cluster deletion) and $x_{ij}=0$ otherwise (Constraint 9). That is, an edge is deleted and not in the solution if $x_{ij}=0$ and $\{i,j\} \in E$. The value for $x_{ij}$ is fixed to zero for all edges that are not element of the input graph (Constraint 10). By maximizing the sum of the weights of the edges remaining in the edited graph, the sum of the weights of the deleted edges is minimized. Constraints 6 to 8 make use of the fact that a cluster graph does not contain any $P_3$s, that is, three vertices being connected by only two edges, thus forming a path. For that reason, the constraints enforce that no $P_3$s exist in the resulting graph. For example, Constraint  6 ensures that if there is an edge between two vertices *i* and *j* and also between *j* and *k*, then there has also to be an edge between vertices *i* and *k*.

By using an ILP formulation, it is possible to retrieve an exact, optimal solution. A major limitation, however, is that, typically, solutions to ILPs have exponential running time with respect to the number of variables, such that with an increase of the number of vertices (i.e., sample size) and/or edges of the input graph it becomes harder that a solution can be found within a reasonable amount of time. Note that practicable sample size, i.e., the sample size for which a solution can be found within a reasonable amount of time, highly depends on the structure of the input graph. This issue can be alleviated when drawing on heuristic approaches to cluster editing problem statements (Hartung & Hoos, 2015). For showcasing cluster editing methods, we focus on optimal solutions retrieved from an ILP formulation. The ILP can be solved using solvers like Gurobi (Gurobi Optimization, LLC, 2019), CPLEX (IBM Corp., 2016), or lp_solve (Berkelaar et al., 2020).

## Workflow and Illustration

**Fig. 3:**
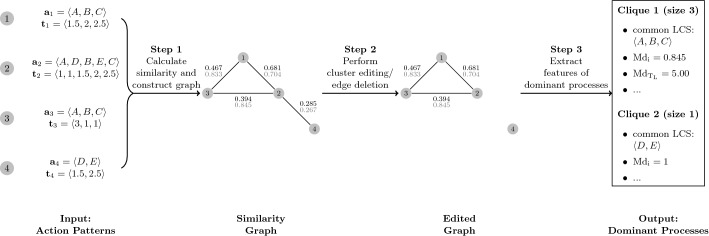
Illustration of the proposed method’s workflow. The numbers on each edge of the depicted graphs give the original similarity measure proposed by Banerjee and Ghosh (2001) (in black) and the modified measure (in gray)

We illustrate the workflow and use of the approach based on the action patterns of the four hypothetical examinees depicted in Fig. 1. The overall workflow is given in Fig. 3. Action patterns are taken as input for determining the similarity of response processes and constructing a weighted graph (Step 1). We provide a step-by-step calculation of the similarity measure proposed by Banerjee and Ghosh (2001) for Examinee 1 and 2, with action patterns ${\mathbf {p}}_1 = [(\text {A},1.5)(\text {B},2)(\text {C}, 2.5)]$ and ${\mathbf {p}}_2 = [(\text {A},1)(\text {D},1)(\text {B},1.5) (\text {E},1) (\text {C},2.5)]$. The times to action associated with the examinees’ $\hbox {LCS}_{12} = \langle \text {A, B, C}\rangle$ are given by ${\mathbf {t}}^1_{l^1(m)} = \langle 1.5, 2, 2.5\rangle$ and ${\mathbf {t}}^2_{l^2(m)} = \langle 1,1.5,2.5\rangle$. The average similarity of times to action associated with $\text {LCS}_{12}$ is $\text {sim}_{\hbox {LCS}_{12}} = \frac{1}{3} \left( \frac{1}{1.5}+\frac{1.5}{2}+\frac{2.5}{2.5} \right) = 0.805$. The geometric mean of the action sequences’ importance is $\left( \frac{6}{6}\frac{5}{7}\right) ^{\frac{1}{2}} = 0.845$, yielding a total similarity of $0.805 \cdot 0.845 = 0.681$. The modified similarity measure is given by $\frac{1+1.5+2.5}{1.5+2+2.5} \cdot 0.845 = 0.704$. Recall that edge weights below a pre-defined threshold $\kappa$ may be set to zero when constructing the graph, incorporating researchers’ beliefs on meaningful similarity.

For identifying dominant response processes, a cluster editing method is performed on the weighted graph (Step 2). In the given example, there are two $P_3$s, (1, 2, 4) and (2, 3, 4). Applying cluster edge deletion leads to deleting the edge connecting action patterns of Examinees 2 and 4 for resolving both $P_3$s and forming two mutually exclusive cliques, (1, 2, 3) and (4). To assess dominant response processes, researchers may extract features of the cliques’ action patterns (Step 3). Possible features of interest may be the most common LCS and the associated average times to action as well as more aggregated features such as the median time-wise importance of the LCS (denoted with $\hbox {Md}_\text {i}$ in Fig. 3), or median time spent on the LCS (denoted with $\hbox {Md}_{\text {T}_\text {L}}$ in Fig. 3).

## Empirical Example

### Data

We illustrate the approach on action patterns from the PIAAC 2012 US sample, focusing on Item U01a, and show how it can be used to distinguish between different strategies that lead to a correct response. In total, there were 678 examinees with a correct response. Since for the whole sample no optimal solution could be found to the ILP within a reasonable amount of time (that is, less than three days) and it is unforeseeable when a solution is to be found, we focused on a randomly chosen subset of $N=225$ examinees, i.e., a third of the original sample.

Item U01a asks examinees to sort emails according to their content into already existing folders. The first action ("start") was dropped since it is by definition associated with a time to action of zero for all examinees. Actions that are not essential to successfully solve the task were aggregated into higher-level categories (e.g., "responding to an email", "seeking help", "keystrokes", "creating new folder", "using the toolbar", "opening folders"). This resulted in 36 aggregated actions in total. An explanation of these actions, proportions of examinees using these actions at least once, as well as median times to action associated with these actions are given in Table 1.

**Table 1:** Description and frequency of performable actions

Action code	Description	Occurrence	Usage	$\hbox {Md}_{\text {TTA}}$
View_Mail*	Opening an email	1841	1.00	2.78
Next	Proceeding to the next task	257	1.00	6.46
Next_OK	Confirming to proceed to the next task	225	1.00	4.37
M_Drag*	Dragging an email	813	0.93	3.85
M_Drop*	Dropping an email	752	0.92	1.78
View_Folder	Opening a folder	756	0.56	2.75
Next_Cancel	Canceling to proceed to the next task	32	0.13	8.94
Toolbar_E_Move*	Moving an email using the toolbar	78	0.10	4.60
Move_E_OK	Confirming to move an email using the toolbar	72	0.09	2.34
Tools	Using tools such as searching or sorting	37	0.06	5.00
Keystroke	Performing keystrokes	86	0.05	0.52
Respond	Writing or responding to an email	45	0.05	3.42
Help	Seeking help	20	0.05	9.00
Move_E_Cancel	Canceling moving an email using the toolbar	2	0.01	4.59
New_Folder	Actions related to creating a new folder	6	0.00	1.62

Occurrence and usage give the total number of occurrence and the proportion of action patterns containing the respective action at least once, respectively. $\hbox {Md}_{\text {TTA}}$ gives the median times to action in seconds. Actions marked with * also include information on which emails were viewed or moved as well as the original and target folder. This information was preserved in analysis but is not displayed in the description due to reasons of item security.

### Analysis

To illustrate (a) how additionally considering action-level times supports a more fine-grained assessment of response processes as well as (b) how different similarity measures determine how detailed dominant response processes are described, we analyzed the data using three similarity measures in separate analyses. We considered both the original similarity measure proposed by Banerjee and Ghosh (2001) as well as the modified similarity measure. Recall that the modified similarity measure takes differences in timing into account but is less sensitive to time-wise differences in single actions. As a baseline for comparison, we considered a similarity measure solely based on actions, neglecting differences in timing. For the latter, we calculated the geometric mean of the length of the LCS relative to the lengths of the action sequences $|{\mathbf {a}}_{i}|$ and $|{\mathbf {a}}_{j}|$ for each pair of action patterns as

11 $$\begin{aligned} s_{{ij}} = \left( \frac{|\hbox {LCS}_{ij}|}{|{\mathbf {a}}_{i}|} \frac{|\hbox {LCS}_{ij}|}{|{\mathbf {a}}_{j}|} \right) ^{\frac{1}{2}}. \end{aligned}$$

Conceptually, this corresponds to the average importance as in Eq. 2, with each action having equal weight, regardless of the associated times to action. In all three analyses, we set $\kappa =.50$, corresponding to considering a medium similarity to be sufficient for action patterns to be in the same clique.^1^

Solutions for the cluster edge deletion problem were computed using Gurobi (Gurobi Optimization, LLC, 2019) through Python version 3.8.1 (Python Software Foundation, 2019). The Python script is available in the supplements. On a 64-bit machine with four cores clocked at 3.6 GHz and 64 GB RAM, Gurobi found an optimal solution for 225 examinees within approximately two hours.

### Results

**Fig. 4:**
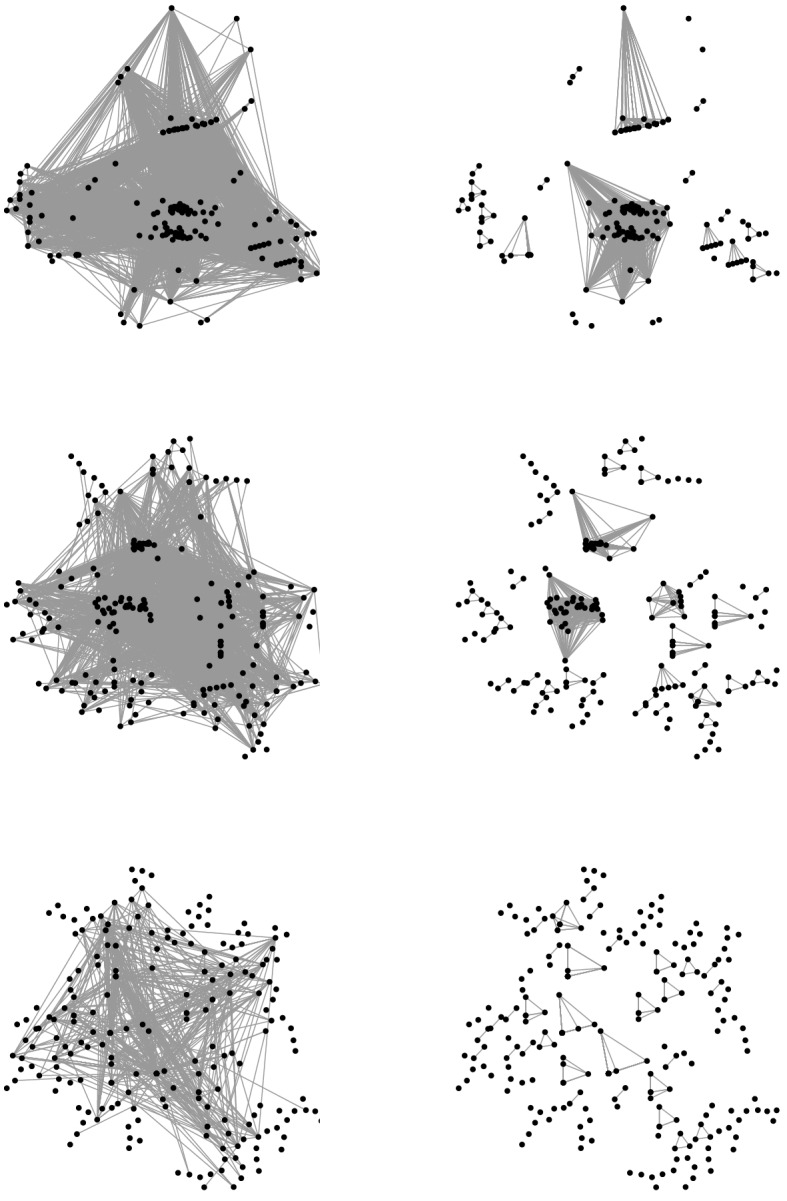
Similarity graph of action patterns to a PIAAC PSTRE task before (left) and after (right) cluster edge deletion. Graphs for the action-based similarity measure, the modified similarity measure and the original similarity measure are given in the upper, middle and lower panels, respectively. A threshold of $\kappa =0.5$ was used in the construction of all three input graphs. Note that for each similarity measure, vertices are placed at the same position in both graphs, such that modifications are visible. Distances between vertices are not proportional to edge weights

Figure 4 gives the similarity graphs prior to and after cluster edge deletion. The input similarity graph based on the action-based similarity measure comprised 15,385 edges, out of which 60.57% were deleted in the cluster edge deletion procedure. The modified and original similarity measures yielded input graphs with 4,577 and 1,006 edges out of which 70.48% and 73.06% were deleted, respectively. The edited graphs consisted of 30, 73, and 140 cliques when being based on the action-based similarity measure, the modified similarity measure, and the original similarity measure, respectively. When employing the action-based similarity measure, only 5.78% of action patterns formed cliques of size one. When considering action-level times, 17.33% and 48.44%, respectively, of action patterns formed cliques of their own. Hence, higher sensitivity of the similarity measures led to less dense input graphs, entailed more edge deletions, and yielded more and smaller cliques. Conceptually, this corresponds to higher differentiation of response processes.

**Fig. 5:**
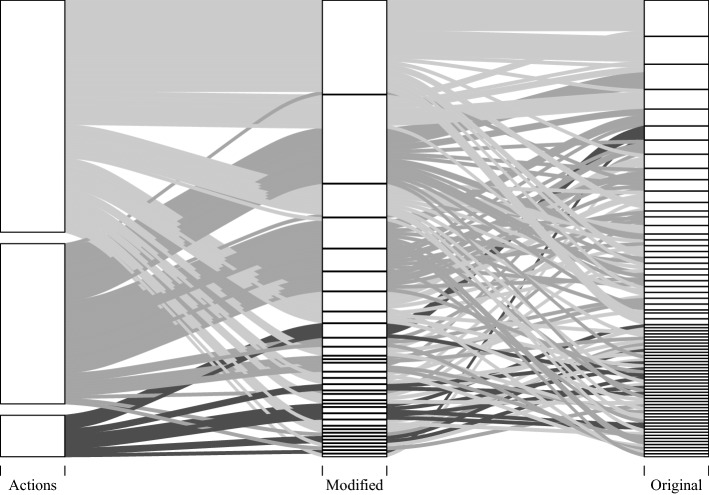
Alluvial plot illustrating clique composition for the different similarity measures. Depicted are only cliques based on action patterns forming the three largest cliques for the action-based similarity measure. These are characterized by moving emails in the order of their appearance using drag-and-drop (Clique 1a), moving emails in the order of their relevance using drag-and-drop (Clique 1b), and moving emails in the order of their appearance using the toolbar (Clique 1c). Note that the alluvial plot does not depict results of a hierarchical clustering procedure but rather results for three independently performed clustering procedures, taking graphs with different similarity measures as input. The similarity measure determines the degree of detailedness with which response processes are described

Figure 5 follows the action patterns constituting the three largest cliques obtained from the action-based similarity measure (76.89% of the analyzed subset) as they are being partitioned to cliques based on similarity measures that take action-level times into account. Increasing the sensitivity to action-level times lead to a further differentiation of captured dominant response processes. This becomes evident from the fact that action patterns tended to get split up into smaller cliques. Thereby, more sensitive similarity measures may uncover differences in response processes that are otherwise not captured. In some cases, action patterns assumed to be governed by a dominant response process when using a less sensitive similarity measure are identified as idiosyncratic response patterns when using a more sensitive measure. Such cases occur when action patterns considerably differ in the timing of actions.

The original similarity is highly sensitive to time-wise differences. This sensitivity may be a useful feature to uncover meaningful differences in response processes. In the present example, however, it entailed a high number of edge deletions on an already less densely connected input graph and, as such, a number of cliques too high to be considered substantial complexity reduction. In the description of dominant response processes captured by the different similarity measures, we therefore focus on the modified similarity measure. We contrast the insights that can be gained on the basis of this measure against the purely action-based measure.

#### Investigating Dominant Response Processes

For illustrating differences in similarity measures, we focused on the three largest cliques identified when using the action-based similarity measure and investigated how these were further differentiated when using the modified time-related similarity measure. For these differentiated processes, only results of cliques of at least size five are displayed. A description of dominant response processes is displayed in Table 2. We assessed the captured response process in terms of the most common LCSs of action patterns constituting the cliques, the proportion of action patterns containing the respective sequence, its median (time-related) importance $\hbox {Md}_\text {i}$, as well as the median time spent on the LCS $\hbox {Md}_{\text {T}_\text {L}}$. In addition, we give the median time spent on task $\hbox {RT}_T$ and median action sequence length $|\mathbf {a}|$. For item security, we do not display information on the actions constituting the most common LCSs.

The three most dominant response processes captured by the purely action-based similarity measure were characterized by the order in which emails were opened and sorted as well as in how emails were moved.^2^ As shown in Table 2, examinees with action patterns constituting Cliques 1a and 3a commonly opened and sorted emails in order of their appearance. In contrast, examinees with action patterns constituting Clique 2a commonly opened Emails 1 and 4 first. Emails 1 and 4 have titles from which it becomes directly evident where the emails need to be sorted to, suggesting that examinees with such action patterns scanned email titles and sorted emails with relevant titles first. While examinees from Cliques 1a and 2a moved emails via drag-and-drop, examinees from Clique 3a used the toolbar. Using the toolbar requires performing more actions than drag-and-drop events, resulting in longer median action sequences and longer time spent on task for Clique 3a as compared to Cliques 1a and 2a. Note that median importance of the common LCS was below 1 in all cliques, indicating that commonly other actions besides those constituting the most common LCS were performed. In most cliques, the most common LCS was sufficient to solve the task at hand, such that other performed actions predominantly concerned exploration of the task environment or performing checks.

Additionally considering action-level times further differentiated between examinees with different strategies. In the following, we exemplarily focus on action patterns characterized by sorting emails in order of their appearance via drag-and-drop. When using the modified time-related similarity measure, four cliques of at least size five were identified. The cliques, however, differed in the time required for the execution of this strategy, with an almost fourfold difference between the groups of examinees requiring the smallest (36 s) and highest (131 s) amount of time for sorting emails via drag-and-drop. Examinees with action patterns constituting Cliques 1b and 2b commonly spent approximately one minute on the task. While examinees from Clique 1b spent this time predominantly sorting emails, examinees from Clique 2b sorted emails faster, but used more time for performing actions that are not essential for solving the task (e.g., exploring the task environment or performing checks). These differences constitute additional features of the response processes that cannot be uncovered on the basis of performed actions or time spent on task alone. Similar effects could be observed for the differentiation of action patterns characterized by sorting emails in order of their relevance.

The original similarity measure proposed by Banerjee and Ghosh (2001) even further differentiated between action patterns. For instance, similar action patterns were partitioned to different cliques based on differences in timing required for each single action involved in dragging emails. As stated above, in the present example, this led to excessively fine-grained descriptions of response processes.

#### Investigating Idiosyncratic Response Processes

To investigate action patterns forming cliques of size one, one may compare time spent on task, action sequence length, and the occurrence of actions not necessary for completing the task of action patterns forming cliques of size one and those describing the dominant response processes. Exemplarily, we do so for cliques identified on the basis of the modified similarity measure. With a median of 175.83 s and ranging from 61.56 to 1062.54 s, time spent on task was commonly longer and more variable for action patterns forming cliques of size one as compared to those forming cliques of at least size five (median: 74.16 s, ranging from 37.54 to 193.56 s). This was also true for action sequence length, ranging from 16 to 137 with a median of 33 for action patterns forming cliques of size one, and from 13 to 36 with a median of 18 for action patterns forming cliques of at least size five. In addition, action patterns forming cliques of their own were characterized by higher incidences of actions unnecessary for successfully completing the task. For instance, 87.18% of action patterns forming cliques of size one contained actions related to opening folders (not compulsory actions to successfully solve the item) in contrast to only 38.40% of action patterns forming cliques of at least size five. Similar patterns could be observed, among others, for actions related to responding to or writing emails (15.38% as compared to 0%), seeking help (17.95 % as compared to 0%), or canceling to proceed to the next task (41.03% as compared to 5.60%). When using the purely action-based similarity measure, capturing idiosyncraticity only in terms of the types and order of performed action, these differences were even more pronounced.

**Table 2:** Description of dominant response processes captured by the action-based and the modified time-related similarity measure

Strategy	Clique, Size	PV	Age	$\hbox {RT}_T$	$|\mathbf {a}|$	%	$\hbox {Md}_\text {i}$	$\hbox {Md}_{\text {T}_\text {L}}$
*Action-based similarity measure*								
Order of appearance, drag-and-drop	1a (84)	309 (27)	33 (13)	68 [59; 88]	19 [16; 24]	0.82	0.74	45 [38; 63]
Relevance, drag-and-drop	2a (58)	309 (24)	42 (12)	85 [69; 105]	18 [15; 22]	0.98	0.72	56 [41; 72]
Order of appearance, toolbar	3a (15)	307 (22)	45 (14)	148 [117; 212]	28 [20; 32]	0.73	0.68	88 [68; 106]
*Modified time-related similarity measure*								
Order of appearance, drag-and-drop	1b (34)	321 (22)	32 (10)	61 [53; 72]	17 [16; 21]	0.79	0.80	48 [44; 52]
	2b (12)	315 (24)	27 (9)	59 [46; 67]	20 [18; 22]	0.83	0.67	36 [35; 38]
	3b (7)	313 (25)	35 (8)	64 [61; 79]	17 [17; 18]	0.86	0.81	57 [50; 58]
	4b (5)	286 (22)	50 (14)	157 [148; 176]	24 [18; 26]	0.60	0.70	131 [118; 135]
Relevance, drag-and-drop	5b (32)	305 (23)	39 (14)	79 [68; 94]	15 [14; 18]	0.69	0.79	64 [57; 68]
	6b (11)	315 (26)	41 (11)	79 [75; 93]	24 [20; 27]	0.45	0.65	61 [55; 64]
	7b (11)	305 (19)	48 (10)	117 [110; 129]	17 [16; 19]	0.91	0.79	92 [85; 98]
	8b (8)	315 (28)	30 (7)	65 [54; 77]	21 [18; 26]	0.75	0.70	44 [40; 47]
Order of appearance, toolbar	9b (5)	316 (18)	45 (16)	112 [110; 123]	19 [18; 23]	1.00	0.93	102 [93; 110]

PV: plausible value mean and standard deviation; age: mean and standard deviation of age; $\hbox {RT}_T$: time spent on task median and middle 50% range; $|\mathbf {a}|$: action sequence length median and middle 50% range; %: percentage of action patterns forming the respective clique that contain the most common LCS; $\hbox {Md}_\text {i}$: median importance of the most common LCS, refers only to action patterns containing the LCS; $\hbox {Md}_{\text {T}_\text {L}}$: median time spent on most common LCS, refers only to action patterns containing the LCS.

#### Relating Response Processes to External Variables

Process data contain much richer information on examinee behavior and should, as such, better differentiate between examinees than the mere fact whether they were able to solve a given task (see Tang, Wang, Liu, & Ying, 2020). For investigating whether the detected response processes indeed contain more detailed information on overall performance, we investigated the mean and standard deviation of PSTRE plausible values within each response process group.^3^ In addition, we illustrate how the response processes can be further investigated by relating them to additional covariates. We here focused on age, which in previous studies (e.g., Tang, Wang, Liu, & Ying, 2020) has been shown to be related to both differences in performed actions and time spent on task. Note that due to the small size of some cliques, differences between the cliques need to be interpreted with caution.

As evidenced in Table 2, dominant response processes identified solely based on performed actions were not predictive of examinees’ proficiency levels. That is, in general, examinees with different proficiencies did not favor one of the three identified dominant strategies. Considering action-level times, however, supported further differentiating between examinees with different proficiency levels. Within each strategy group, examinees spending less time on the most common LCS also tended to have higher proficiency levels. A similar effect could be observed for age. Considering action-level times supported further differentiating between examinees, with older examinees typically requiring more time for executing the actions constituting the most common LCS. This becomes especially evident from Clique 4b, being characterized by both the highest median time spent on the most common LCS and the highest mean age.

We corroborated our conclusions based a second randomly chosen subset. The found dominant response processes are summarized in “Appendix.” Results concerning dominant response processes were, by and large, comparable. There were, however, differences in PVs between examinees with response processes identified solely based on performed actions, and differences in PVs and age between groups of response processes identified based on the modified similarity measure were less pronounced than in the results described here.

## Discussion

The method presented in this paper is (to our knowledge) the first to consider both actions and the associated action-level times to distinguish between different response processes. To this end, we integrated methods from clickstream analysis and graph-modeled data clustering with psychometrics. For quantifying the degree of similarity of action patterns, two similarity measures were provided that take both the specific actions performed as well as action-level times into account. Cluster edge deletion supports retrieving homogeneous, interpretable, well-separated cliques of action patterns, each describing a response process. The similarity measure as well as the cluster editing method can easily be customized and adapted to achieve different degrees of detailedness of the captured response processes. Below, we provide guidelines to that end.

Note that for addressing psychometric research objectives, the method is not restricted to the application to action patterns associated with a certain type of response but might very well be employed to investigate action patterns in tests with interactive modes such as collaborative, game- and simulation-based tasks. These rapidly become more widely used (von Davier, Zhu, & Kyllonen, 2017).

Besides presenting an approach that jointly considers actions and action-level times for describing response processes, the present paper showcased cluster editing methods as an innovative approach for clustering data based on similarity measures. In the present context, using cluster edge deletion comes with the following major advantages over other common clustering techniques: First, the number of clusters does not need to be pre-specified. As shown in the empirical example, some action patterns have very low similarity to other action patterns, posing idiosyncratic response processes. Employing cluster editing to classify response processes allows these action patterns to form cliques of their own. Applying techniques where the number of clusters needs to be specified, however, would likely lead to sorting such idiosyncratic action patterns into other clusters, yielding less homogeneous, less separated, and therefore less interpretable clusters. Second, researchers can exert control over what defines a cluster and, as such, over the degree and kind of homogeneity of clusters. Cluster homogeneity may considerably ease interpretation of retrieved results.

In an application of the method to correct responses to a single item from the PIAAC PSTRE domain, we demonstrated how additionally considering action-level times may provide a more fine-grained description of response processes than considering actions alone. We could identify easy-to-interpret, homogeneous, well-separated cliques describing dominant response processes. The identified response processes supported differentiating between examinees that were able to solve the given task and revealed differences in response behavior between examinees of different age. By comparing similarity measures differing in their sensitivity to time-wise differences, it was also showcased how researchers may exert control over the degree of detailedness with which response processes are captured. In the considered data set, the more sensitive similarity measure led to excessively fine-grained descriptions of dominant response processes. This may be of utility in other applications with less variety in performable actions.

### Guidelines and Further Modifications for Controlling the Degree of Detailedness

The presented approach is exploratory in nature. As such, it is recommended to analyze data using several settings and to evaluate whether these yield reasonable results. Nevertheless, the decisions that need to be taken all entail subject-matter interpretations such that researchers also may have some preliminary presumptions informing these decisions. In any case, decisions should be guided by the degree of detailedness researchers aim to achieve in the description of response processes. In the following, we will derive guidelines and discuss further modifications for exerting control over how detailed response processes are depicted.

#### Data Pre-processing

In data processing, in analogy to the empirical example, researchers may aggregate similar actions into higher-level categories (see Eichmann et al., 2020, for a recent example). For instance, if researchers are not interested in *how* examinees moved an email but only want to consider *whether* an email was moved, they may aggregate drag-and-drop events and moving emails via the toolbar into a single category.

#### Similarity Measure

The similarity measure can flexibly be adapted by differently weighing its components. We presented two versions that differ in their sensitivity to differences in the timing of actions within the area of overlap of performed actions. If researchers are especially interested in time spent on specific action types (e.g., drag-and-drop events), they could up-weigh their contribution to the similarity measure. In addition, based on theoretical considerations, researchers might either consider times *to* action—that is, defining the time associated with an action as the time that elapsed from performing the preceding action—or times *from* action—that is, defining the time associated with an action as the time that elapsed between performing the respective action and the subsequent one.

#### Cluster Editing Settings

Cluster editing techniques allow researchers exerting a maximum amount of control over what defines a cluster and, as such, over how broadly response processes are described. First, for identifying broader response process categories, for allowing less similar action patterns to form a clique, the threshold $\kappa$ can be set to lower values. Second, researchers may allow for edge insertion. When doing so, researchers can control the costs of edge insertion. For instance, when researchers want vertices within a clique to show a high degree of similarity to most but not necessarily all of the other vertices, they can allow for edge insertion but make it costly. Third, requirements of what defines a cluster can be relaxed. Examples for relaxed cluster requirements are editing the graph by inducing subgraphs where every vertex has edges to all but at most *s* vertices instead of cliques (Guo, Komusiewicz, Niedermeier, & Uhlmann, 2010; Komusiewicz, 2011) or fixing the maximum number of modifications incident with a vertex (Komusiewicz & Uhlmann, 2012).

### Limitations and Future Research

The proposed method is exploratory in nature, and further research is needed on the conditions for retrieving meaningful, generalizable, and valid results. In the empirical example, we could show that the identified response processes allow further differentiating between examinees able to solve a given task. In a similar vein, future research could relate differences in applied response processes to external criteria to assess whether differences in response processes contain meaningful information above and beyond the specific test-taking situation and possess predictive validity for real-world outcomes (see Balart, Oosterveen, & Webbink, 2018; Hitt, Trivitt, & Cheng, 2016; Zamarro, Cheng, Shakeel, & Hitt, 2018, for examples considering test-taking behavior on classical test items). In addition, found solution strategies could be evaluated by subject-matter experts or corroborated by comparing results for similar tasks possibly requiring similar solution strategies (e.g., assessing agreement of results for different mail sorting tasks).

Likewise, such studies could inform guidelines for researchers’ decisions concerning similarity measures and the formulation of the cluster editing problem. Researchers might oftentimes not have sufficient domain knowledge to decide on how to weigh different components contributing to the similarity measure, whether to consider times to or from action, how to set the threshold $\kappa$, and to which to degree to allow for edge insertion. Decisions on these settings can impact the results of the proposed method. For now, when no preliminary presumptions exist, we suggest to take an exploratory approach to these decisions by systematically varying different aspects of the method—such as the similarity measure, the threshold $\kappa$, and whether and to which degree to allow for edge insertion—and checking whether the results obtained correspond to the degree of detailedness researchers want to achieve. More in-depth studies could compare results retrieved from method variations by assessing convergence with expert judgments or by means of cross-validation with tasks with similar requirements. In addition, future research can develop more objective, data- rather than interpretation-driven methods for setting the threshold $\kappa$ in analogy to, e.g., the knee point method for determining the number of clusters in settings that require for pre-determining the number of clusters (e.g., Salvador & Chan, 2004).

As it is the case with other common methods for investigating response processes (e.g., Greiff et al., 2015; Hao et al., 2015; He, Liao, & Jiao, 2019b; Wang et al., 2020), the proposed method is focused on assessing response processes to a single item. For investigating whether examinees with a similar response process on a given task also approach other tasks in a similar manner, researchers may separately analyze multiple tasks and then visually assess clique composition across tasks using alluvial plots. In addition, the cluster editing toolbox entails methods that allow for partitioning multilayer graphs and, as such, pose more sophisticated means to identifying subgroups with similar behavioral patterns across the whole test. Cluster editing for multilayer graphs is performed on a set of graphs consisting of the same vertex set, called layers, and aims to transform all layers into only slightly differing cluster graphs (Chen, Molter, Sorge, & Suchy, 2018). Investigating the utility of these methods for investigating response processes to multiple tasks is a promising topic for future research.

A major challenge with solving cluster editing problems is their possibly unfeasible running time when applied to action patterns of larger samples while aiming at retrieving an optimal solution. Depending on the structure of the input graph, maximum sample sizes can vary. In biological applications, for instance, larger data sets with 4,000 instances could be solved within a reasonable amount of time (Hartung & Hoos, 2015). Hence, although the sample size for which a solution could be obtained within a feasible amount of time was quite small in the empirical example, this must not necessarily be the case for other interactive tasks that yield differently structured input graphs. Although the currently available solvers were too slow for solving our data set, the continuous development of algorithms for cluster editing methods (see, e.g., Tsur, 2019; van Bevern, Froese, & Komusiewicz, 2018) gives rise to the hope for reasonably efficient solvers in the near future. Moreover, establishing action patterns as a common application for cluster editing might trigger a similar development as seen for clustering biological data, with researchers developing new solvers focusing on data sets from common applications which in turn leads to faster algorithms (see Hartung & Hoos, 2015, and the references therein). Until then, when researchers aim at retrieving an exact solution, we recommend to reduce complexity of the input graph, e.g., by setting higher threshold values, thereby deleting a higher number of edges prior to analysis and reducing connectivity of the input graph. Note, however, that doing so may result in smaller cliques and there may be only minor differences in response processes described by some cliques. In addition, heuristic cluster editing algorithms could be applied (see, e.g., Livne et al., 2015; Wittkop et al., 2007). It should, however, be noted that solutions found by heuristic cluster editing algorithms are not necessarily optimal and there is no way of telling whether found solutions are optimal or how far from optimal the found solutions are. Note that this is also true for other clustering methods such as the canonical *k*-means algorithm. To alleviate this issue, researchers may draw on a combination of heuristic and exact cluster editing methods, investigating the trustworthiness of heuristics by comparison with an exact solution for a smaller subsample.

## Appendix: Corroboration of Dominant Response Processes

See Table 3.

**Table 3:** Cross-validation: description of dominant response processes captured by the action-based and the modified time-related similarity measure for a second randomly chosen subsample of $N=225$

Strategy	Clique, Size	PV	Age	$\hbox {RT}_T$	$|\mathbf {a}|$	%	$\hbox {Md}_\text {i}$	$\hbox {Md}_{\text {T}_\text {L}}$
*Action-based similarity measure*								
Order of appearance, drag-and-drop	1av (88)	312 (27)	34 (13)	85 [69; 119]	18 [16; 22]	0.70	0.73	51 [37; 68]
Relevance, drag-and-drop	2av (48)	305 (26)	39 (13)	100 [66; 129]	19 [16; 23]	1.00	0.70	60 [39; 84]
Order of appearance, toolbar	3av (16)	293 (34)	30 (11)	85 [64; 124]	26 [23; 36]	0.44	0.64	37 [28; 50]
*Modified time-related similarity measure*								
Order of appearance, drag-and-drop	1bv (37)	308 (26)	37 (14)	73 [62; 90]	16 [14; 18]	0.68	0.84	65 [56;75]
	2bv (20)	302 (30)	39 (14)	108 [100; 126]	16 [14; 17]	0.80	0.81	79 [76; 90]
	3bv (12)	323 (31)	31 (12)	63 [54; 72]	20 [17; 24]	0.50	0.74	50 [48; 51]
	4bv (9)	306 (25)	29 (12)	70 [59; 79]	20 [18; 25]	1.00	0.82	51 [48; 65]
	5bv (5)	310 (20)	40 (18)	145 [139; 169]	21 [21; 21]	0.80	0.79	124 [114; 128]
Relevance, drag-and-drop	6bv (21)	313 (29)	38 (9)	67 [57; 87]	18 [16; 23]	1.00	0.74	45 [41; 48]
	7bv (8)	302 (21)	40 (14)	113 [100; 116]	28 [25; 32]	0.75	0.68	66 [61; 71]
	8bv (5)	305 (19)	30 (13)	109 [77; 128]	22 [17; 28]	0.80	0.63	60 [57; 61]

PV: plausible value mean and standard deviation; age: mean and standard deviation of age; $\hbox {RT}_T$: time spent on task median and middle 50% range; $|\mathbf {a}|$: action sequence length median and middle 50% range; %: percentage of action patterns forming the respective clique that contain the most common LCS; $\hbox {Md}_\text {i}$: median importance of the most common LCS, refers only to action patterns containing the LCS; $\hbox {Md}_{\text {T}_\text {L}}$: median time spent on most common LCS, refers only to action patterns containing the LCS. Note that in the second randomly chosen subset, examinees moving emails via the toolbar were partitioned to cliques smaller than 5 when using the modified similarity measure.

## Footnotes

1 We additionally analyzed subsamples of the data with $\kappa =.45$ and $\kappa =.55$. These settings mainly led to subsuming, respectively, further differentiation, of smaller cliques but did, by and large, not affect conclusions concerning dominant response processes captured on the basis of each similarity measure.

2 Note that for all cliques displayed, proportions of action patterns containing the most common LCS were below 1. This indicates that the most common LCS of each clique did not occur in all of its constituting action patterns. Nevertheless, since all action forming a clique are required to display a certain degree of similarity to each other, action patterns that did not contain the whole most common LCS still showed strong overlap.

3 The PSTRE performance is defined by four levels: below Level 1 (0–240), Level 1 (241–290), Level 2 (291–340), and Level 3 (341–500). For more details, refer to OECD (2013).
